# Review of the sawfly genus *Empria* (Hymenoptera, Tenthredinidae) in Japan

**DOI:** 10.3897/zookeys.150.1968

**Published:** 2011-11-28

**Authors:** Marko Prous, Mikk Heidemaa, Shinohara Akihiko, Villu Soon

**Affiliations:** 1 Department of Zoology, Institute of Ecology and Earth Sciences, University of Tartu, Vanemuise 46, 51014 Tartu, Estonia; 2 Department of Zoology, National Museum of Nature and Science, 4-1-1 Amakubo, Tsukuba-shi, Ibaraki, 305-0005 Japan

**Keywords:** Sawflies, new species, new synonymy, key, cytochrome c oxidase I, internal transcribed spacer

## Abstract

The following eleven *Empria* species are reported from Japan: *Empria candidata* (Fallén, 1808), *Empria japonica* Heidemaa & Prous, 2011, *Empria liturata* (Gmelin, 1790), *Empria loktini* Ermolenko, 1971, *Empria plana* (Jakowlew, 1891), *Empria quadrimaculata* Takeuchi, 1952, *Empria rubicola* Ermolenko, 1971, *Empria tridens* (Konow, 1896), *Empria tridentis* Lee & Ryu, 1996, *Empria honshuana* Prous & Heidemaa, **sp. n.**, and *Empria takeuchii* Prous & Heidemaa, **sp. n.** The lectotypes of *Poecilosoma pallipes* Matsumura, 1912, *Empria itelmena* Malaise, 1931, *Tenthredo candidata* Fallén, 1808, and *Tenthredo* (*Poecilostoma*) *hybrida* Erichson, 1851 are designated. *Empria itelmena* Malaise, 1931, **syn. n.** is synonymized with *Empria plana* (Jakowlew, 1891). *Poecilosoma pallipes* Matsumura, 1912, previously assigned to *Empria*, is transferred to *Monsoma*, creating *Monsoma pallipes* (Matsumura, 1912), **comb. n.** Results of phylogenetic analyses using mitochondrial (COI) and nuclear (ITS1 and ITS2) sequences are also provided.

## Introduction

With 51 valid species-level taxa (Taeger et al. 2010; Prous et al. 2011b), *Empria* Lepeletier & Serville, in Latreille et al. 1828 is one of the largest genera in the Allantinae. Nevertheless, it still remains rather poorly studied in comparison with other tenthredinid sawflies. *Empria* species are often misidentified because of the lack of easily observable diagnostic characters. Fortunately, their genitalia frequently possess clear differences even between closely related species mostly enabling their reliable identification. Though the knowledge on most of the European *Empria* species can be regarded as satisfactory (Zhelochovtsev and Zinovjev 1988; Prous et al. 2011b), very little is known about Eastern Palaearctic species. According to Takeuchi (1952a), more than seven *Empria* species had been found in Japan, but most of them remained unidentified. Until recently, only two species had been identified (Takeuchi 1952a; b; Abe and Togashi 1989), and one of them, *Empria pallipes* (Matsumura 1912), actually belongs to *Monsoma* MacGillivray, 1908 (see results). Prous et al. (2011b) reported three additional species. Here we report 11 species from Japan, two of them described as new. One male, probably representing a new *Empria* species (sp. 1) is also discussed but not yet described as new due to insufficient material.

No attempts to reconstruct the phylogeny of *Empria* have been made so far. Some preliminary results based on a limited number of species can be found in Prous et al. (2011b), which focuses on the *Empria longicornis* species group. Only few intrageneric groups have been proposed, which might be monophyletic. In particular, *Empria* is sometimes divided into the subgenera *Parataxonus* MacGillivray, 1908 [now comprising *Empria candidata* (Fallén, 1808) and *Empria multicolour* (Norton, 1862)] and *Empria* s. str. (all other species) (Ross 1936; Zhelochovtsev and Zinovjev 1988; 1996; Yan et al. 2009). Within *Empria* s. str., the *Empria hungarica* (Konow, 1895) (see Heidemaa and Viitasaari 1999) and the *Empria longicornis* (Thomson, 1871) species groups (see Prous et al. 2011b) have been proposed. In addition, the *Empria immersa* species group can be defined for the species possessing highly similar penis valves, which have a characteristic long apical spine (Smith 1979; Zhelochovtsev and Zinovjev 1988; Prous et al. 2011b). To examine the phylogenetic relationships within *Empria* based on DNA sequences, we here expand the dataset of Prous et al. (2011b) by including 7 more species (six outside and one inside of the *longicornis*-group). For this, we use one continuous mitochondrial region (full COI, two complete, and one incomplete tRNAs) and one nuclear region (ITS1 and ITS2 within the rRNA locus) analysed separately and in combination using Bayesian methods.

## Material and methods

Pinned specimens studied are from the following institutional collections:

BMNH Natural History Museum, London, United Kingdom (G. Broad, N. Dale-Skey Papilloud, S. Ryder, N. Springate);


CSCS Central South University of Forestry and Technology, Changsha, China (M.-C. Wei);


DEI Senckenberg Deutsches Entomologisches Institut, Müncheberg, Germany (A. Taeger, S. M. Blank, A. D. Liston);


EIHU Hokkaido University, Sapporo, Japan (M. Suwa);


HNHM Hungarian Natural History Museum, Budapest, Hungary (S. Csősz, L. Zombori);


NHRS Naturhistoriska Riksmuseet, Stockholm, Sweden (H. Vårdal);


NSMT National Museum of Nature and Science, Tokyo, Japan (A. Shinohara);


SIZ I. I. Schmalhausen Institute of Zoology, National Academy of Sciences of Ukraine, Kiev, Ukraine (I. N. Pavlusenko);


TUZ Zoological Museum of the University of Tartu, Estonia (J. Luig);


UOPJ Osaka Prefecture University, Sakai, Japan (T. Hirowatari);


USNM National Museum of Natural History, Smithsonian Institution, Washington DC, USA (D. R. Smith);


UUZM Uppsala University, Museum of Evolution, Uppsala, Sweden (H. Mejlon);


YUIC Yeungnam University Insect Collections, Gyeongsan, South-Korea (J.-W. Lee);


ZISP Zoological Institute of the Russian Academy of Sciences, St. Petersburg, Russia (S. Belokobylskij, A. Zinovjev);


ZMH Zoological Museum, Helsinki, Finland (P. Malinen);


ZML Museum of Zoology and Entomology, Lund University, Lund, Sweden (R. Danielsson);


ZMUC Zoological Museum of the University, Copenhagen, Denmark (L. Vilhelmsen).


Specimens from the private collections of Erik Heibo, Guy T. Knight, and of the second author (MH) were also studied.

For morphological analyses, male penis valves, female lancets (valvula 1), and external characters of the adults were studied.

To dissect the penis valves, genital capsules were separated from the specimen and macerated in KOH or NaOH (10–15%) for 6–12 hours at room temperature, or treated with proteinase K using High Pure PCR Template Preparation Kit (Roche, Mannheim) and following manufacturer's protocol.

Imaging methods are described in Prous et al. (2011b). All images made for this study are deposited in the Morphbank database (http://www.morphbank.net/?id=592670).

**Figures 1–2.**
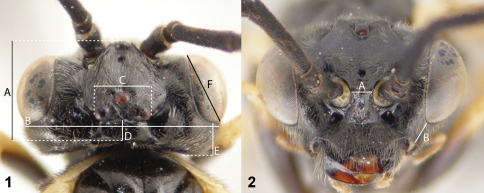
Distances measured on the head capsule. **1** *Empria quadrimaculata*, head in dorsal view, female (NSMT083) (A, head length, B, head breadth, C, breadth of the postocellar area, D, length of the postocellar area, E, minimal distance between the eye and the occipital carina = head length behind the eye, F, length of the eye) **2** *Empria quadrimaculata*, head in anterior view, female (NSMT083) (A, minimal distance between toruli, B, malar space).

Morphological terminology follows Viitasaari (2002). To differentiate between species, some distances were measured on the head capsule (Prous et al. 2011b): maximal lengths of flagellomeres, head length (Fig. 1A), head breadth behind the eyes (Fig. 1B), length between lateral margins of lateral ocelli (Fig. 1C; “breadth of postocellar area"), length of the postocellar area (Fig. 1D), head length behind the eye in dorsal view (Fig. 1E; head positioned with posterior margins of lateral ocelli and eyes aligned), length of the eye (Fig. 1F), length between toruli (antennal sockets) (Fig. 2A), maximal and minimal length of the temple (http://www.morphbank.net/?id=781392), and the length of malar space (Fig. 2B; from here on referred to as “malar space").

For molecular phylogenetic analyses, DNA sequences of the internal transcribed spacers 1 and 2 (ITS1 and ITS2), and a mitochondrial DNA (mtDNA) fragment containing tRNA-Cys, tRNA-Tyr, cytochrome c oxidase I (COI), and partial tRNA-Leu, were obtained using methods described in Prous et al. (2011b). However, because amplification of ITS2 of *Empria honshuana* sp. n. failed using the primers CAS5p8sFc and CAS28sB1d (Ji, Zhong and He 2003; Prous et al. 2011b), we used the primers AM1 (5´ TGT GAA CTG CAG GAC ACA TGA 3´) and AM2 (5´ATG CTT AAA TTT AGG GGG TAG TC 3´) (Marinucci et al. 1999; Heidemaa et al. 2004) instead. The PCR programme in this case consisted of an initial denaturing step at 95°C for 1 min, followed by 43 cycles of 20 s at 95°C, 30 s at 65–55°C (a touchdown profile was used, in which the annealing temperature decreased from 65°C to 55°C by 0.5°C every cycle), and 70 s at 68°C; the last cycle was followed by a final 7 min extension step at 68°C. For some older air-dried museum specimens, it was possible to obtain the sequences only partially. Sequences reported here have been deposited in the GenBank (NCBI) database (accession numbers JN029842–JN029898). As suggested by Chakrabarty (2010), DNA sequences from type material are here referred to as genetypes.

Boundaries of the sequenced tRNA and ITS2 genes were identified as described by Prous et al. (2011b). Phylogenetic analyses of ITS genes were performed using Bali-Phy 2.0.2 (Suchard and Redelings 2006) since this program has implementations to handle difficult-to-align sequences. In order to enhance the speed of calculation, sequences were aligned manually for detecting and fixing the conserved positions prior to analysis with Bali-Phy. Four independent analyses were run (203 213–262 061 iterations) using the GTR + I + G[4] model. The first 10 000–60 000 iterations were discarded as “burn in" after examination of log-likelihood scores in Tracer 1.4 (available from http://beast.bio.ed.ac.uk/Tracer).

Phylogenetic analysis of the mitochondrial genes and combined analysis of the nuclear and mitochondrial genes were performed with MrBayes 3.1.2 (Huelsenbeck and Ronquist 2001; Ronquist and Huelsenbeck 2003) using the GTR + I + G[4] model. Mitochondrial sequences were aligned manually, and prior to phylogenetic analyses, non-coding and ambiguously aligned tRNA regions, one insertion of three base pairs in COI of *Monsoma pulveratum* (Retzius, 1783), and two to three amino acid coding codons of COI at the 3´ end (the last three codons of *Empria quadrimaculata* and *Empria rubicola* could not be unambiguously aligned with the last two codons of other species) were excluded. In the combined analysis we used MAP (maximum a posteriori) alignment of ITS obtained from one of the four analyses with Bali-Phy. Both mitochondrial and combined datasets were run for 5 000 000 MCMC generations, with trees and lnL's sampled at intervals of 100 generations. The first 25% of generations were discarded as “burn-in". *Monsoma pulveratum* was used to root the trees.

**Figures 3–6.**
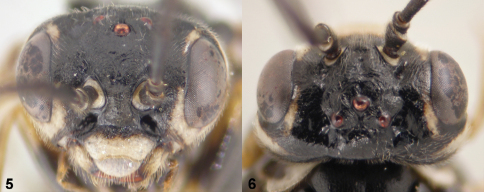
**3** *Monsoma pallipes*, habitus in dorsal view, female (NSMT174) **4** *Empria candidata*, habitus in dorsal view, female (NSMT187) **5** *Empria candidata*, head in anterior view, female (NSMT208) **6** *Empria candidata*, head in dorsal view, female (NSMT208).

## Data resources

The data underpinning the analyses reported in this paper are deposited in the Dryad Data Repository at doi: 10.5061/dryad.fs262s48 (Prous et al. 2011a) and at GBIF, the Global Biodiversity Information Facility, http://ipt.pensoft.net/ipt/resource.do?r=japanese_empria.

## Results

### Key to Japanese *Empria* and *Monsoma* (imagines)

**Table:** 

1	Abdominal terga without pale insulated (detached) paired patches (Fig. 3); length of postocellar area more than 3.5 times diameter of lateral ocellus; first flagellomere 0.9–1 times as long as flagellomeres 2–3 combined; propleura meeting broadly in front; on hind wing cross-vein m-cu present, cell M closed; valvula 1 as in Fig. 13; Hokkaido [East Palaearctic]	*Monsoma pallipes*
–	Abdominal terga with pale, more or less insulated paired patches (Fig. 4); length of postocellar area less than 3.0 times diameter of lateral ocellus; first flagellomere 0.4–0.7 times as long as flagellomeres 2–3 combined; propleura not meeting or meeting only narrowly in front; on hind wing cross-vein m-cu present or absent, cell M closed or open	*Empria* 2
2	At least facial orbits dorsally and part of temples pale (Fig. 5–6); clypeus flat without median keel; on hind wing cross-vein m-cu absent, cell M open; claws simple or with minute subbasal tooth; number of serrulae 18–21, valvula 1 as in Fig. 14; posterior margin of sternum 9 in male notched (Fig. 7), penis valve as in Fig. 25; Hokkaido [Holarctic]	*Empria candidata*
–	Facial orbits and temples black (Fig. 1–2, 9–10); clypeus with median keel (distinct mostly in anterior part of clypeus only); on hind wing cross-vein m-cu usually present, cell M usually closed; claws variable; number of serrulae 13–18(19); posterior margin of sternum 9 in male rounded (Fig. 8); penis valve different	3
3	female	(female of *Empria* sp. 1 is currently unknown) 4
–	male	13
4	Postocellar area (1.9)2.1–2.5 times wider than long (Fig. 1), trochanters and trochantelli black; serrulae as in Fig. 15–16; abdominal terga with 2–3 pairs of pale patches	*Empria quadrimaculata* group 5
–	Postocellar area 1.5–2.1 times wider than long (Fig. 9–10) and / or trochanters and trochantelli pale; serrulae different (Fig. 17–24); abdominal terga with 2–6 pairs of pale patches	6
5	Abdominal terga mostly with 2 pairs of pale patches; antennae long, flagellum mostly 2.1–2.5 times longer than head breadth; in most specimens flagellomeres 1 and 2 about equally long; number of serrulae 17–19 (Fig. 15); cannot always be distinguished morphologically from *Empria rubicola*; Honshu, Shikoku, Kyushu	*Empria quadrimaculata*
–	Abdominal terga mostly with 3 pairs of pale patches; antennae short, flagellum mostly 1.9–2.2 times longer than head breadth; in most specimens flagellomere 1 longer than flgm. 2; number of serrulae 16–18 (Fig. 16); cannot always be distinguished morphologically from *Empria quadrimaculata*; Hokkaido [also Sakhalin Oblast, Russia]	*Empria rubicola*
6	Malar space 2.2–2.5 times longer than lateral ocellus diameter and abdominal terga with 5–6 pairs of large pale patches; claws bifid; clypeus in most specimens at least distally pale; tegulae pale; serrulae as in Fig. 17; Hokkaido, Honshu (Yamagata) [East Palaearctic]	*Empria plana*
–	Malar space 1.5–2.0 times longer than lateral ocellus diameter and abdominal terga with 2–6 pairs of small or large pale patches or malar space 1.9–2.2 times longer than lateral ocellus diameter and abdominal terga with 3 pairs of small pale patches; claws with small subbasal tooth or simple; clypeus black; tegulae black or pale; serrulae different	7
7	Serrulae as in Fig. 22–24; length of head 2.3–2.9 (2.5–3.2 in *Empria tridens*) times greater than length of head behind eyes (Fig. 9); trochanters and trochantelli black or slightly pale	(*Empria japonica*, *Empria loktini*, *Empria tridens*) 11
–	Serrulae as in Fig. 18–21; length of head 2.9–3.3 times greater than length of head behind eyes (Fig. 1, 10) and / or trochanters and trochantelli pale	8
8	Trochanters and trochantelli pale; tegulae completely pale	9
–	Trochanters and trochantelli black; tegulae mostly black	10
9	Flagellum 2.2–2.4 times longer than breadth of head; abdominal terga with 3 pairs of small pale patches (Fig. 11); serrulae as in Fig. 18; Hokkaido, Honshu [East Palaearctic]	*Empria tridentis*
–	Flagellum 1.8–2.0 times longer than breadth of head; abdominal terga with 3–4 pairs of large pale patches (Fig. 12); serrulae as in Fig. 19; Hokkaido, Honshu	*Empria takeuchii*
10	Basal serrulae conspicuously protruding (Fig. 20); claws simple or with minute subbasal tooth; abdominal terga with 5–6 pairs of pale patches; Hokkaido [Palaearctic]	*Empria liturata*
–	Basal serrulae not conspicuously protruding (Fig. 21); claws with conspicuous subbasal tooth; abdominal terga with 4 pairs of pale patches; Honshu	*Empria honshuana*
11	Flagellum 2.5–2.7 times longer than breadth of head; maximal length of temple 1.40–1.55 times greater than minimal length of temple; serrulae as in Fig. 23; Hokkaido	*Empria japonica*
–	Flagellum 1.8–2.3 times longer than breadth of head; maximal length of temple less than 1.35 times greater than minimal length of temple; serrulae as in Fig. 22, 24	12
12	Abdominal terga mostly with 5 pairs of pale patches; number of serrulae 16–18 (Fig. 22); Hokkaido [Palaearctic]	*Empria tridens*
–	Abdominal terga mostly with 2–3 pairs of pale patches; number of serrulae 13–14(15) (Fig. 24); Hokkaido [also Sakhalin Oblast, Russia] … *Empria loktini*
13	Postocellar area (2.1)2.2–2.5 times wider than long and trochanters and trochantelli black; penis valves as in Fig. 26–27	*Empria quadrimaculata* group 14
–	Postocellar area 1.7–2.1(2.2) times wider than long or trochanters and trochantelli at least partly pale; penis valves as in Fig. 28–36	15
14	Valviceps with small basal lobe, ventroapical part of valviceps slightly bent towards its basal part (Fig. 26); flagellum 2.9–3.3 times longer than breadth of head; in most specimens flagellomere 7 not distinctly shorter than length of eye; Honshu, Shikoku, Kyushu	*Empria quadrimaculata*
–	Valviceps with large basal lobe, ventroapical part of valviceps strongly bent towards its basal part (Fig. 27); flagellum 2.6–3.0 times longer than breadth of head; in most specimens flagellomere 7 distinctly shorter than length of eye; Hokkaido [also Sakhalin Oblast, Russia]	*Empria rubicola*
15	Valviceps with long apical spine (Fig. 28); malar space 1.9–2.3 times longer than lateral ocellus diameter; Hokkaido, Honshu (Yamagata) [East Palaearctic]	*Empria plana*
–	Valviceps without long apical spine (Fig. 29–36); malar space 1.3–1.8 times longer than lateral ocellus diameter	16
16	Trochanters, trochantelli, and tegulae pale; abdominal terga mostly with 3 pairs of pale patches	17
–	Trochanters black; trochantelli black or with barely visible median pale band or patch; tegulae black or pale; abdominal terga with 2–5 pairs of pale patches	18
17	Valviceps with large dorsobasally pointing spine at dorsoapical part (Fig. 29); postocellar area 1.9–2.3(2.4) times wider than long; flagellum 2.6–3.7 times longer than breadth of head; Hokkaido, Honshu [East Palaearctic]	*Empria tridentis*
–	Valviceps with small dorsally pointing tooth at dorsoapical part (Fig. 30); postocellar area 2.0–2.7 times wider than long; flagellum 2.2–2.7 times longer than breadth of head; Hokkaido, Honshu	*Empria takeuchii*
18	Antennae short, flagellum 2.3–3.0 times longer than breadth of head	19
–	Antennae long, flagellum 3.2–3.8 times longer than breadth of head	22
19	Valviceps with large dorsoapical spine (Fig. 31–32)	20
–	Valviceps with small dorsoapical tooth (Fig. 33–36)	21
20	Dorsal margin of valviceps concave (Fig. 31); claws with minute subbasal tooth; abdominal terga with (2)3–4 pairs of pale patches; Honshu	*Empria honshuana*
–	Dorsal margin of valviceps convex (Fig. 32); claws simple or with minute subbasal tooth; abdominal terga with 5 pairs of pale patches; Hokkaido [Palaearctic]	*Empria liturata*
21	Apical part of valvular duct extending clearly further from dorsal rim of valvura (Fig. 33); abdominal terga mostly with 2–3 pairs of pale patches; Hokkaido [also Sakhalin Oblast, Russia]	*Empria loktini*
–	Apical part of valvular duct reaching almost the dorsal rim of valvura or extending only slightly further from it (Fig. 34); abdominal terga mostly with 4–5 pairs of pale patches; Hokkaido [Palaearctic]	*Empria tridens*
22	Basal lobe of valviceps short, valviceps less than 0.65 as long as valvura (Fig. 35); maximal length of temple (1.30)1.35–1.50 times greater than its minimal length; Hokkaido	*Empria japonica*
–	Basal lobe of valviceps long, valviceps more than 0.8 as long as valvura (Fig. 36); maximal length of temple less than 1.35 times greater than its minimal length; Hokkaido	*Empria* sp. 1

## Taxonomy

### Monsoma pallipes

(Matsumura, 1912) comb. n.

http://species-id.net/wiki/Monsoma_pallipes

Poecilosoma pallipes Matsumura, 1912: 61–62.

#### Type locality.

Japan, Hokkaido, Sapporo. Lectotype **(here designated)** female (Fig. 37), EIHU. Labelled: “Maruyama 5/24", “7", “Poecilosoma pallipes Mats., Type".

#### Taxonomic affinities.

*Monsoma pallipes* can most easily be differentiated from the other *Monsoma* species, *Monsoma pulveratum* (Retzius, 1783), *Monsoma inferentium* (Norton, 1868), and *Monsoma faustum* Zhelochovtsev, 1961, by the colouration of the head capsule: temples, genae, facial orbits, paraantennal field laterally, and area between toruli and lateral to median ocellus are pale brown in *Monsoma pallipes*, while in the other three species the head capsule is black.

#### Host plants.

Unknown, but could be associated with *Alnus* as for *Monsoma pulveratum* and *Monsoma inferentium* (Smith 1979; Pieronek 1980; Chevin 2004).

#### Distribution.

East Palaearctic. Specimens studied are from Japan (Hokkaido) and Russia (Primorsky Krai).

#### Notes.

Male unknown. Matsumura (1912) did not give the number of specimens he used for the original description. A female syntype bearing a red type label is hereby designated as the lectotype.

**Figures 7–12.**
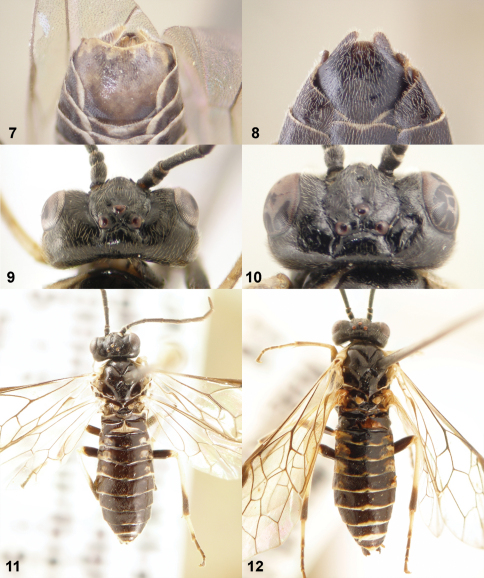
**7** *Empria candidata*, posterior tip of the abdomen in ventral view, male (TUZ282970) **8** *Empria quadrimaculata*, posterior tip of the abdomen in ventral view, male (NSMT228) **9** *Empria loktini,* head in dorsal view, female (NSMT014) **10** *Empria honshuana* sp. n., head in dorsal view, female paratype (NSMT-Hym2011-2-3-4) **11** *Empria tridentis*, habitus in dorsal view, female (NSMT051) **12** *Empria takeuchii* sp. n.,habitus in dorsal view, female paratype (NSMT032).

### Empria candidata

(Fallén, 1808)

http://species-id.net/wiki/Empria_candidata

Tenthredo candidata Fallén, 1808: 105–106. **Type locality.** Sweden. Lectotype (**here designated**) female [in good condition], UUZM. Labelled: “Uppsala Univ. Zool. Mus. Typsamlingen nr. 1940b Hymenoptera Tenthredo candidata Fallén 1808" [red, printed, partially handwritten], “♀" [pale, handwritten], “LECTOTYPUS 2008 [printed part, red label] TENTHREDO CANDIDATA FALLÉN 1808 Des. M.Heidemaa & M.Prous [handwritten part]", “*Empria* 2008 *candidata* (Fallén, 1808) ♀ M.HEIDEMAA & M.PROUS" [white, printed]. 3 paralectotype females of *Tenthredo candidata* designated (“PARALECTOTYPUS 2008 [printed part, red label] TENTHREDO CANDIDATA FALLÉN 1808 Des. M.Heidemaa & M.Prous" [handwritten part]) belong in *Empria immersa* (Klug, 1818) [nr. 1940a], *Empria pumila* (Konow, 1896) [nr. 1940c], and *Empria fletcheri* (Cameron, 1878) [nr. 1940d] (respectively labelled by M. Heidemaa & M. Prous).
Tenthredo (Allantus) repanda Klug, 1816: 77–78.

#### Taxonomic affinities.

The morphologically closest species is the Nearctic *Empria multicolor*, from which *Empria candidata* can be distinguished by the following characters: femora predominantly and most other parts of legs at least partly black (legs are almost entirely yellowish in *Empria multicolor*), tarsal claws simple or with a minute inner tooth (with a long subbasal tooth in *Empria multicolor*), shallowly emarginated clypeus (deeply emarginated in *Empria multicolor*), and postocellar area more than 1.6 times wider than long (less than 1.5 in *Empria multicolor*) (see also Smith 1979).

#### Host plants.

*Betula* (Lorenz and Kraus 1957; Verzhutskii 1981), *Betula pendula* Roth (under the name *Betula verrucosa* in Verzhutskii 1966).

#### Distribution.

Holarctic. Specimens studied are from China (Heilongjiang), Estonia, Finland, Japan (Hokkaido), Russia (Kamchatka Krai, Khabarovsk Krai, Leningrad Oblast, Primorsky Krai), South-Korea, Sweden, Switzerland, United Kingdom, USA (Maine).

**Figures 13–16.**
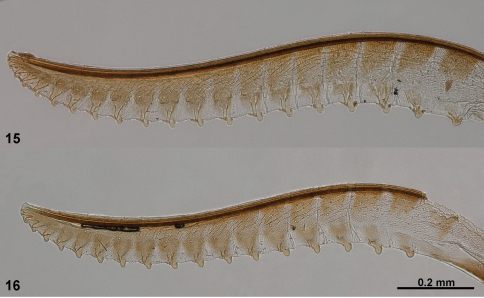
Lancets (valvulae 1) of *Monsoma* and *Empria*. **13** *Monsoma pallipes* (NSMT173) **14** *Empria candidata* (NSMT208) **15** *Empria quadrimaculata* (NSMT155) **16** *Empria rubicola* (USNM2051678_053).

### Empria japonica

Heidemaa & Prous, 2011

urn:lsid:zoobank.org:act:BA25596E-802D-43E3-B351-52A0BAB1B78F

http://species-id.net/wiki/Empria_japonica

Empria japonica Heidemaa & Prous in Prous et al. 2011b: 22–24. Type locality: Japan, Hokkaido, Ginsendai, Kamikawa-chô, 43°40'N, 143°01'E, 947 m, selectively cut forest. Holotype female, NSMT.

#### Genetype accessions in GenBank.

USNM2051678_019: HM177347 (hologenetype COI), HM177397 (hologenetype ITS1), HM177299 (hologenetype ITS2); USNM2051678_009: HM177346 (paragenetype COI), HM177396 (paragenetype ITS1), HM177298 (paragenetype ITS2); USNM2051678_003: HM177345 (paragenetype COI), HM177395 (paragenetype ITS1), HM177297 (paragenetype ITS2).

#### Taxonomic affinities.

Belongs to *Empria longicornis* group (see Prous et al. 2011b). Morphologically the most similar species are *Empria tridens* (Konow, 1896), *Empria longicornis*, and *Empria* sp. 1, from which *Empria japonica* can be distinguished by having maximal length of temple mostly more than 1.40 (in males rarely 1.30) times greater than minimal length of temple (less than 1.35 in the other three species). *Empria* sp. 1 differs clearly also by its penis valve (cf. Fig. 35–36).

#### Host plants.

Unknown, but could be *Rubus idaeus* L. subsp. *melanolasius* (Dieck) Focke (see Prous et al. 2011b).

#### Distribution.

Japan (Hokkaido).

**Figures 17–20.**
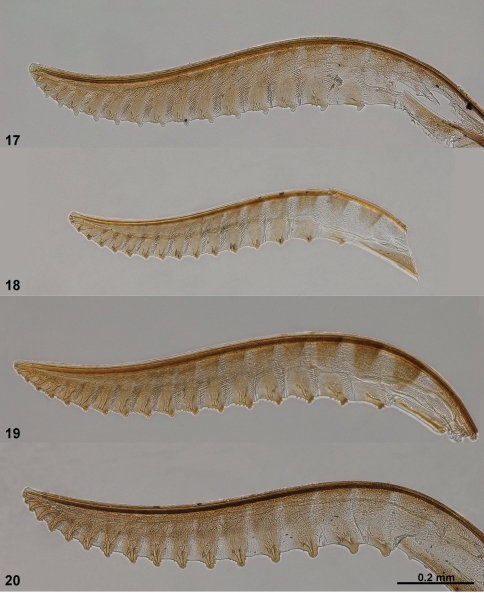
Lancets (valvulae 1) of *Empria*. **17** *Empria plana* (NSMT026) **18** *Empria tridentis* (USNM2051678_013) **19** *Empria takeuchii* sp. n., holotype (NSMT044) **20** *Empria liturata* (USNM2051678_054).

### Empria honshuana

Prous & Heidemaa sp. n.

urn:lsid:zoobank.org:act:AF95BFA0-C12F-46AB-B50B-8A8CA18B34CF

http://species-id.net/wiki/Empria_honshuana

#### Type-locality.

Japan, Honshu, Tochigi Prefecture, Bicchuzawa, Bato, Nakagawa.

#### Holotype.

1 female, NSMT. Labelled: “[JAPAN: Honshu] Bicchuzawa, Bato, Nakagawa, Tochigi 13. IV. 2006 S. Ibuki", “NSMT110", “Holotypus ♀ *Empria honshuana* spec. nov. design. : M. Prous & M. Heidemaa 2011", “*Empria honshuana* sp.n. Prous & Heidemaa det. 2011".

#### Paratypes.

“[JAPAN:Honshu] Hikagezawa Mt. Takao-san Tokyo 21. IV. 1996 A. Shinohara", 1 female, NSMT073 (NSMT); “[JAPAN: Honshu] Bicchuzawa, Bato Tochigi Pref. 9. IV. 2005 A. Shinohara" 24 males, NSMT109, NSMT115, NSMT121–137, NSMT166–170 (NSMT), 1 male TUZ615362 (TUZ); “[JAPAN: Honshu] Bicchuzawa, Bato Tochigi Pref. 23. IV. 2005 A. Shinohara" 1 male, NSMT171 (NSMT); “[JAPAN: Honshu] Bicchuzawa, Bato Tochigi Pref. 29. IV. 2005 A. Shinohara" 1 female, TUZ615361 (TUZ); “[JAPAN:Honshu] Annaigawa, nr Mt. Takao-san Tokyo 17. IV. 1994 A.&T.Shinohara" 1 female, NSMT198, 2 males, NSMT120, NSMT200 (NSMT); “[JAPAN:Honshu] Akigase-koen Saitama Pref. 14. IV. 1996 A. Ta., N. & To. Shinohara" 1 female, NSMT204 (NSMT); “[JAPAN: Honshu] Bicchuzawa, Bato Nakagawa, Tochigi 13. IV. 2006 S. Ibuki" 1 male, NSMT106 (NSMT); “[JAPAN:Honshu] Bicchuzawa Bato, Tochigi 1. V. 2010 S. Ibuki" 1 female, NSMT-Hym2011-2-3-4 (NSMT); “JAPAN: Chiba Pref. Okusa-cho, Wakaba-shi 35°36.5'N, 140°11.6E' 23 March 1997 O. S. Flint, Jr." 1 female, USNM2051678_016 (USNM); “JAPAN: Honshu Himuro-machi Utsunomyia-shi Tochiji-ken [Utsunomiya-shi Tochigi-ken], Mal. 2-15.IV.2009, Mal. trap Takeyuki Nakamura leg." 1 male, USNM2057434_04 (USNM).

#### Genetype accessions in GenBank.

NSMT106: JN029870 (paragenetype COI), JN029890 (paragenetype ITS1), JN029854 (paragenetype ITS2); NSMT-Hym2011-2-3-4: JN029891 (paragenetype ITS1); USNM2051678_016: JN029871 (paragenetype COI), JN029892 (paragenetype ITS1).

#### Female.

**Body length.** 6.0–6.9 mm.

**Colour.** Black; following parts unpigmented, pale: apical maxillary palpomeres; posterodorsal margin of pronotum in lateral parts; tegulae (except lateroproximal part); median band or patch of pro-, meso-, and metatrochantellus; profemur apically; protibia in anterior and partly posterior aspects; mesotibia partly in anterior and posterior aspects; metatibia basally; tarsomere 1 of hind leg basally; paired patches on abdominal terga 2–5; at least partially posterior margins of terga (tergum 10 dorsally more widely) and sterna; and cenchri. Labrum from yellowish-brown to blackish.

**Head.** Head behind eyes in dorsal view subparallel sided; postocellar area trapeziform, its length equal to or longer than 2 times diameter of lateral ocellus; distinct and diverging lateral postocellar furrows going from ocelli towards occiput at least to the distance of ocellus diameter; area between frontal crests clearly exceeding the level of crests in dorsal view; postocellar area with indistinct punctures and interspaces, more or less glossy; punctures more regular on temples and postocular area, face with more irregular punctures; wrinkled interspaces more prominent on frontal area; clypeus with rough irregular punctures, more or less fused; ocellar and postocellar area convex, slightly raised; clypeus tridentate with median keel distinct mostly in anterior part of clypeus only, median tooth smaller than lateral teeth; malar space about equal to or shorter than distance between antennal sockets; frontal ridge V-shaped; pit in central part of frontal field present; median ocellus surrounded by groove, with short distinct longitudinal furrow anteriorly, and with similar but mostly less distinct furrow posteriorly. Maximal length of temple 1.2–1.4 times greater than its minimal length; flagellum 1.9–2.0 times longer than breadth of head.

**Thorax.** Mesoscutellum, mesoscutellar appendage, and metapostnotum more or less glossy, almost impuctate or with indistinct shallow punctures; metascutellum with irregular fine punctures; punctures on mesoscutum more evident on lateral and anterior regions of the median lobes, fading towards central regions; mesepisternal punctures variable between specimens, from rather weak with intespaces almost glossy to more distinct with sculptured, interspaces; mesepimeron with setae on posterior part; metepisternum with evenly distributed setae; metepimeron in central part without setae; distance between cenchri 1.1–1.4 times of cenchrus width; wings hyaline, venation brownish, becoming paler near junction to thorax; closed cell M in hindwing present; tarsal claws with conspicuous subbasal tooth.

**Figures 21–24.**
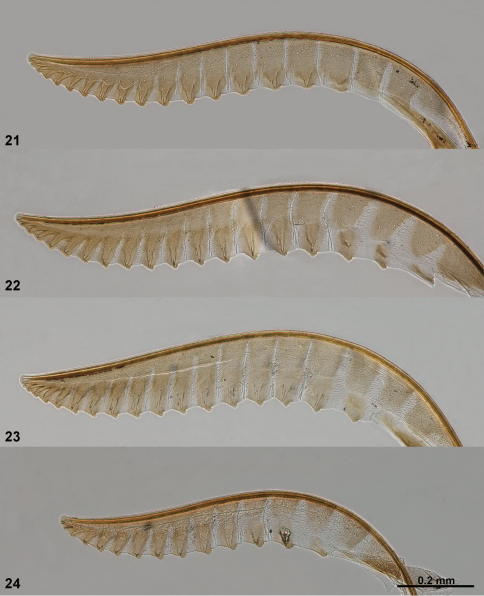
Lancets (valvulae 1) of *Empria*. **21** *Empria honshuana* sp. n., paratype (USNM2051678_016) **22** *Empria tridens* (USNM2051678_018) **23** *Empria japonica*, holotype (NSMT USNM2051678_019) **24** *Empria loktini* (TUZ615180).

**Abdomen.** Terga on most parts with transverse keel-like sculpticells and with short setae (about half of lateral ocellus diameter), sometimes with shallow punctures at median parts of terga 2–4; posterior parts of terga (6) 7–9 (occasionally terga 3–10) at median line with small more or less triangular pale regions; ventral margin of valvula 3 slightly bending towards apex, slightly longer than valvifer 2; serrulae of valvula 1 as in Fig. 21, number of serrulae 15–16.

#### Male.

(Mostly the differences compared to female are given).

**Body length.** 4.8–5.6 mm.

**Colour.** Unpigmented, whitish or yellowish brown: anterolateral (seldom also posterolateral) margins of tegulae; protibia in anterior aspect, often partly also in posterior aspect; mesotibia partly in anterior aspect; outer margins of harpes; and paired patches on abdominal terga 2–(3)/4/(5).

**Head.** Area between frontal crests reaching or slightly exceeding the level of crests in dorsal view; malar space less than or equal to distance between antennal sockets; length of postocellar area about 2 times of lateral ocellus diameter; maximal length of temple 1.25–1.45 times greater than its minimal length; flagellum 2.3–2.6 times longer than breadth of head.

**Thorax.** Distance between cenchri variable, up to 2 times width of cenchrus. Tarsal claws with minute subbasal tooth.

**Abdomen.** Tergum 8 with indistinct tergal hollows which form semioval or semicircular depression reaching 1/3–1/2 of tergum length and sometimes possessing indefinite central procidentia. Posterior margin of sternum 9 round; penis valve as in Fig. 31.

#### Taxonomic affinities.

Based on the similarities in penis valves, the closest species is *Empria sulcata* Wei & Nie, 1998 from China (see http://www.morphbank.net/?id=643394). While the penis valves of both species can easily be distinguished, the distinctly concave dorsal margin of valviceps of these species is a unique characteristic within *Empria*. Serrulae of the two species are clearly different (cf. Fig. 21 and http://www.morphbank.net/?id=700325). Externally the species can mainly be distinguished by colouration: in *Empria sulcata* tegulae are completely pale and legs extensively yellowish, while in *Empria honshuana* tegulae are at least partly and legs predominantly black.

#### Host plants.

Unknown.

#### Distribution.

Japan (Honshu).

#### Etymology.

The species name refers to the type locality, Honshu, the main island of Japan.

### Empria liturata

(Gmelin, 1790)

http://species-id.net/wiki/Empria_liturata

Tenthredo liturata Gmelin, 1790: 2668. Type locality: Europe [type specimens probably lost (Blank et al. 2009: 13)].
Poecilosoma undulata Konow, 1885: 122. Type locality: Czech Republic, Altvater. Syntype female, DEI [examined].

#### Note

See Taeger et al. (2010) for full list of synonyms.

#### Taxonomic affinities.

The most similar species morphologically appears to be Nearctic *Empria ignota* (Norton, 1867). The clearest differences between these species can be seen in the structure of penis valves (Fig. 32; http://www.morphbank.net/?id=694564).

#### Host plants.

*Filipendula ulmaria* (L.) Maxim., *Geum rivale* L. (based on ex ovo rearings by MP in Estonia). *Fragaria vesca* has also been suggested (Enslin 1914), but this requires confirmation.

#### Distribution.

Palaearctic. Specimens studied are from Belgium, Croatia, Czech Republic, Denmark, Estonia, France, Germany, Hungary, Italy, Japan (Hokkaido), Russia (Leningrad Oblast), Switzerland, United Kingdom.

**Figures 25–30.**
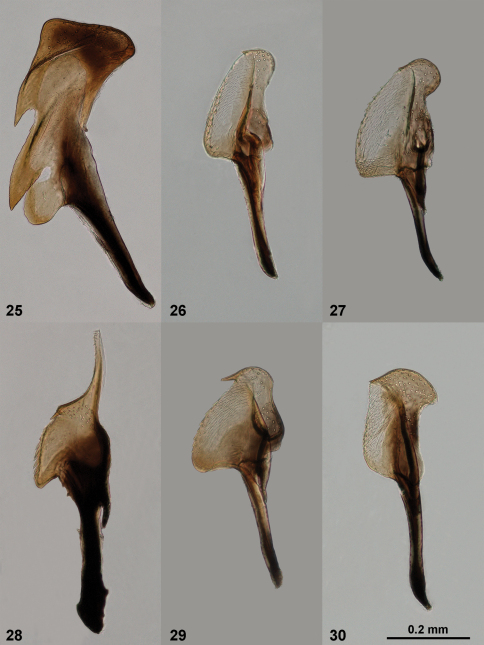
Penis valves of *Empria*. **25** *Empria candidata* (NSMT036) **26** *Empria quadrimaculata* (UOPJ03) **27** *Empria rubicola* (USNM2051678_042) **28** *Empria plana* (NSMT201) **29** *Empria tridentis* (TUZ615182) **30** *Empria takeuchii* sp. n., paratype (NSMT112).

### Empria loktini

Ermolenko, 1971

http://species-id.net/wiki/Empria_loktini

Empria loktini Ermolenko, 1971: 22–23. Type locality: Russia, Sakhalin Oblast, Novoaleksandrovsk. Holotype female, SIZ [examined].

#### Taxonomic affinities.

Belongs to *Empria longicornis* group, morphologically the closest is *Empria basalis* Lindqvist, 1968, which can be distinguished from *Empria loktini* by clearly different penis valves, lancets (see Prous et al. 2011b), and in most cases also by some external differences (in *Empria loktini* metatibia is pale in basal 1/3 and the abdominal terga bear 2–3 pairs of pale patches, in *Empria basalis* metatibia is mostly black and the terga have 4–5 pairs of pale patches).

#### Host plants.

Unknown.

#### Distribution.

East Palaearctic. Specimens studied are from Japan (Hokkaido) and Russia (Sakhalin Oblast).

### Empria plana

(Jakowlew, 1891)

http://species-id.net/wiki/Empria_plana

Tenthredo (Poecilostoma) hybrida Erichson in: Ménétriés in: Middendorff, 1851: 60–61. Primary homonym of *Tenthredo (Tenthredo) hybrida* Eversmann, 1847. Type locality: Udskoj Ostrog [Russia, Khabarovsk Krai, Udskoe]. Lectotype **(here designated)** female, ZISP. Labelled: “Poecilostoma hybrida* Erichs. Midd. R." [pale, handwritten], “Lectotypus ♀ *Tenthredo (Poecilostoma) hybrida* Erichson, 1851 design. : M.Prous & M.Heidemaa 2011" [red, printed], “*Empria plana* (Jakovlev 1891) det. M.Prous 2008" [white, printed].
Poecilosoma plana Jakowlew, 1891: 31. Type locality: Russia, Irkutsk. Holotype female, ZISP [examined].
Empria itelmena Malaise, 1931: 23, **syn. n.** Type locality: Kamtschatka, E[lisowo] [Russia, Kamchatka Krai]. Lectotype **(here designated)** female, NHRS. Labelled: “Kamtschatka Malaise", “E", "Typus", “Lectotypus ♀ *Empria itelmena* Malaise, 1931 design. : M. Prous & M. Heidemaa 2011" [red, printed], “*Empria plana* (Jakovlev 1891) det. M.Prous 2009" [white, printed].
Empria erichsoni Liston, 1995: 241. New name for *Tenthredo (Poecilostoma) hybrida* Erichson, 1851.

#### Taxonomic affinities.

Morphologically the closest species is *Empria immersa* (Klug, 1818), from which *Empria plana* can be distinguished by differences in the structure of serrulae (Fig. 17; http://www.morphbank.net/?id=694567) and penis valves (Fig. 28; http://www.morphbank.net/?id=578888). Externally, the *Empria plana* specimens from mainland Asia differ clearly from *Empria immersa* also by their pale clypeus (black in *Empria immersa*), which is, however, only partly pale or nearly black in Japanese specimens. In this regard, some disagreements concerning the taxonomic status of *Empria plana* should also be noted. Some authors treat this taxon either as a geographical form, or as a subspecies of *Empria immersa* (Verzhutskii 1966; Zhelochovtsev and Zinovjev 1996), but Lindqvist (1972) argues that *Empria plana* (under the name *Empria hybrida* Erichson, 1851) is a separate species (followed also by Taeger et al. 2010). Because of the above mentioned differences between these two taxa, we concur with Lindqvist (1972) in treating them as distinct species. Such conclusion is supported also by current nuclear sequence data (Fig. 38).

#### Host plants.

Possibly *Salix* sp., see Verzhutskii (1966; 1981)under the name *Empria immersa*.

#### Distribution.

East Palaearctic. Specimens studied are from Japan (Hokkaido, Honshu), Mongolia, and Russia (Amur Oblast, Irkutsk Oblast, Kamchatka Krai, Khabarovsk Krai, Primorsky Krai).

**Figures 31–36.**
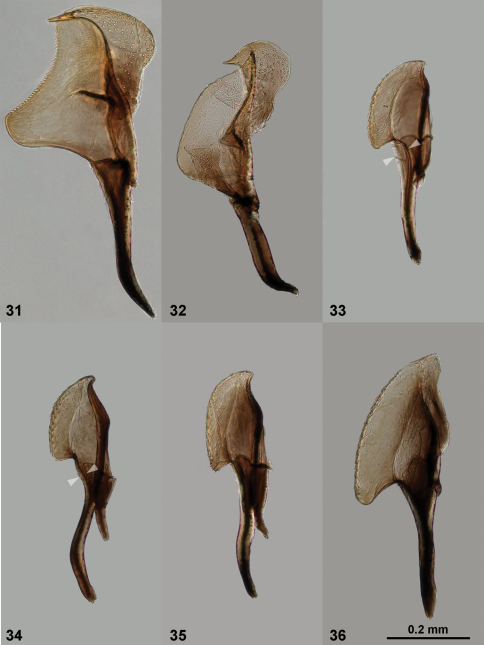
Penis valves of *Empria*. **31** *Empria honshuana* sp. n., paratype (NSMT200) **32** *Empria* *liturata* (USNM2051678_051) **33** *Empria* *loktini* (NSMT105) **34** *Empria tridens* (USNM2051678_024) **35** *Empria japonica*, paratype (NSMT009) **36** *Empria* sp. 1 (USNM2051678_040). The arrowheads illustrate the different position of valvular duct (upper right arrowhead) relative to the dorsal rim of valvura (lower left arrowhead) in *Empria loktini* (Fig. 33) and other species of *longicornis*-group (Fig. 34).

### Empria quadrimaculata

Takeuchi, 1952

http://species-id.net/wiki/Empria_quadrimaculata

Empria quadrimaculata Takeuchi, 1952b: 49–50. Type locality: Japan, Kyoto, Ushio. Holotype female, UOPJ [examined].

#### Taxonomic affinities.

The closest species are *Empria zhangi* Wei & Yan, 2009 (China) and *Empria rubicola* Ermolenko, 1971. *Empria zhangi* (two females and two males studied, including the holotype) can be distinguished from *Empria quadrimaculata* mainly by the following two characters: 1) in female malar space clearly less than two times of the lateral ocellus diameter (about two times in *Empria quadrimaculata* and *Empria rubicola*), in male equal or slightly less than the ocellus diameter (clearly longer in *Empria quadrimaculata* and *Empria rubicola*); and 2) in female flagellum about 2.0 times longer than breadth of head (2.1–2.5 times in *Empria quadrimaculata*), in male 2.4–2.5 times (2.9–3.3 times in *Empria quadrimaculata*). *Empria rubicola* has shorter antennae and three pairs of pale patches (mostly two in *Empria quadrimaculata*) on terga. The penis valves of *Empria zhangi* and *Empria quadrimaculata* are very similar (http://www.morphbank.net/?id=693502; Fig. 26), while *Empria rubicola* can be distinguished from the two by relatively large basal lobe of the valviceps and by the ventroapical part clearly bent towards its basal part (Fig. 27). Valvula 1 appears indistinguishable in all three species.

#### Host plants.

Okutani (1954) indicated *Geum japonicum* Thunb., but noted later that the specific identity of the reared *Empria* species was uncertain (Okutani 1967).

#### Distribution.

Japan (Honshu, Shikoku, Kyushu).

### Empria rubicola

Ermolenko, 1971

http://species-id.net/wiki/Empria_rubicola

Empria rubicola Ermolenko, 1971: 21–22. Type locality: Russia, Sakhalin Oblast, Novoaleksandrovsk. Holotype female, SIZ [examined].

#### Taxonomic affinities.

The closest species are *Empria zhangi* and *Empria quadrimaculata* (see under *Empria quadrimaculata* Takeuchi, 1952 for details).

#### Host plants.

Unknown. Holotype female and the studied paratypes (1 female, 2 males) were collected from *Rubus idaeus* L. subsp. *melanolasius* (Dieck) Focke(under the name *Rubus sachalinensis* in Ermolenko 1971), which is a common plant in Hokkaido.

#### Distribution.

East Palaearctic. Specimens studied are from Japan (Hokkaido) and Russia (Sakhalin Oblast). Most probably this species has to be removed from the list of Chinese species (Yan et al. 2009), because *Empria rubicola* has clypeus and upper half of the mesepisternum black (not yellow brown) and abdominal terga 2–4 (not 2–6) each with a pair of pale patches.

### Empria takeuchii

Prous & Heidemaa sp. n.

urn:lsid:zoobank.org:act:BDE02124-C81A-4705-91F4-34B40134B0C1

http://species-id.net/wiki/Empria_takeuchii

#### Type-locality.

Japan, Honshu, Yamanashi Prefecture, Utsukushinomori, Yatsugatake Mts.

#### Holotype.

1 female, NSMT. Labelled: “[JAPAN:Honshu] Utsukushinomori 1500–1700m Yatsugatake Mts. Yamanashi Pref. 5–8. VI. 2000 A. Shinohara", “NSMT044", “Holotypus ♀ *Empria takeuchii* sp. n. design. : M. Prous & M. Heidemaa 2011", “*Empria takeuchii* sp.n. Prous & Heidemaa det. 2011".

#### Paratypes.

“Shimashima Nagano Pref 16. V. 1984 A. Shinohara", 1 female, NSMT032 (NSMT); “[JAPAN:Honshu] Kamiange, Mt. Jinba Tokyo 27. IV. 2003 A. Shinohara", 1 male, NSMT037 (NSMT); “Ōmi, Ō hara [Ōhara] Kyoto Pref. 15. V. 1984 R. Inagawa", 1 female, NSMT041 (NSMT); “[Ōmi, Ōhara] Sakyo-ku, Kyoto Kyoto Pref. May, 14, 1984 T. Matsumoto leg." 1 female, NSMT211 (NSMT); “[JAPAN: Honshu] Yokotemichi, ca. 850m 35-22-39N 133-31-21E Mt. Daisen Tottori Pref. 28-29. IV. 2007 A. Shinohara“, 1 male, NSMT112 (NSMT); “Takihata Kawachi-Nagano Osaka 22. IV. 1981 A. Shinohara", 1 male, NSMT213 (NSMT); “JAPAN: Ishikawa Pref., Mt. Shiritaka 637 m, May 19 1979 D. Smith & I. Togashi" 1 female, USNM2051678_047 (USNM); “JAPAN: Honshu Tamozawa, Nikkô-shi Tochigi-ken, Mal. trap 13-27.iv.2009 Takeyuki Nakamura leg.", 1 male, USNM2057434_03 (USNM).

#### Other material examined.

“JAPAN, Hokkaido Ginsendai, Kamikawa-chô 43°40'N, 143°01'E, 947 m Selectively cut forest 6–27.vi.2008 Mal. trap, A. Ueda leg" 1 female, USNM2051678_011 (USNM); “JAPAN, Hokkaido Sekihoku-tôge, Kamikawa-chô, natural forest, 993 m 43°40'N, 143°06'E, 6–27.vi.2008 Mal. trap, A. Ueda leg." 3 males, USNM2051678_008, USNM2051678_031, USNM2051678_061 (USNM); “42°57'N,141°14'E Hakken-zan Sapporo, Hokkaidō JAPAN 16.v.2009 Takuma YOSHIDA leg." 2 males, USNM2057434_06, USNM2057434_07 (USNM).

#### Female.

**Body length.** (5.1)6.4–6.9 mm.

**Colour.** Black; following parts more or less unpigmented, whitish or yellowish brown: labrum; apical maxillary and labial palpomeres; tegulae completely; posterodorsal margin of pronotum in lateral part rather widely, upper part of posterolateral margin of pronotum quite narrowly; pro-, meso-, and metacoxa apically; pro-, meso-, and metatrochanter partly or in most part; pro-, meso-, and metatrochantellus partly or completely; profemur in anterior, posterior, and lateral aspects; mesofemur and metafemur apically slightly; protibia in anterior and posterior aspects; mesotibia in most part; metatibia in basal 2/3; tarsomere 1 of hind leg in basal 2/3; paired patches on abdominal terga 2–4(5); posterior margins of terga and sterna; and cenchri (in one female only posterior margin).

**Head.** Head behind eyes in dorsal view subparallel sided; postocellar area trapeziform, its length mostly less than or equal to 2 times of lateral ocellus diameter; area between frontal crests in dorsal view reaches or slightly exceeds the level of crests; face and clypeus with somewhat irregular punctures, less shining compared to vertex and especially to postocellar area; ocellar and postocellar area at least slightly raised; clypeus tridentate, with median tooth smaller than lateral teeth; clypeus with median keel; malar space (minimal ventro-ocular distance) shorter or equal to distance between antennal sockets; frontal ridge “V"-shaped, central part of frontal field with distinct pit; maximal length of temple 1.25–1.4 times greater than its minimal length; flagellum 1.8–2.0 times longer than breadth of head.

**Thorax.** Anterior part of mesoscutum with more or less distinct punctures, its median and postero-lateral portions in most part with sparse indistinct punctures and glossy interspaces, or almost impunctate, glossy; mesoscutellum, mesoscutellar appendage, and metapostnotum impunctate and glossy; mesepisternum with more or less indistinct punctures, mostly glossy; mesepimeron with setae on posterior part; metepisternum with evenly distributed setae; metepimeron in central part without setae; distance between cenchri in most specimens about equal to cenchrus width, but sometimes slightly greater; wings hyaline with brownish venation; closed cell M in hindwing present; tarsal claws with conspicuous subbasal tooth.

**Abdomen.** Terga mostly with keel-like (sometimes mixed with scale-like) sculpticells and short setae (about half of lateral ocellus diameter); ventral margin of valvula 3 abruptly bending towards apex, about equal in length to valvifer 2; serrulae of valvula 1 as in Fig. 19, number of serrulae (15)16–17.

#### Male.

(Mostly the differences compared to female are given).

**Body length.** 5.6–5.8 mm.

**Colour.** Unpigmented, whitish or yellowish are: meso- and metatrochanter apically; pro-, meso-, and metatrochantellus partly; mesofemur only apically, or in anterior, posterior, and lateral aspects; metafemur apically; mesotibia partly in anterior, posterior, and lateral aspects, or in most part; metatibia in basal 1/3 or in basal 1/2; outer margins of harpes; paired patches on abdominal terga 2–4(3).

**Head.** Area between frontal crests in dorsal view not exceeding the level of crests; length of postocellar area 1.5–2.0 times of lateral ocellus diameter; maximal length of temple 1.25–1.45 times greater than its minimal length; flagellum 2.2–2.7 times longer than breadth of head.

**Abdomen.** Posterior margin of sternum 9 round; tergum 8 without tergal hollows and procidentia; penis valve as in Fig. 30.

#### Taxonomic affinities.

Morphologically, no certain closest relative can be specified. Superficially may resemble *Empria rubicola* (based on males), *Empria honshuana* (based on females), or *Empria tridentis* (both have pale trochanters and trochantelli). Penis valve (Fig. 30) and valvula 1 (Fig. 19) clearly distinguish this species from all other known species of *Empria*. According to the molecular analyses (of ITS1 and ITS2 combined with mtDNA sequences), the closest species are those of the *Empria longicornis* and *Empria immersa* species groups, and *Empria tridentis* (Fig. 38, 40).

#### Host plants.

Unknown.

#### Distribution.

Japan (Hokkaido, Honshu).

#### Etymology.

The specific name refers to Kichizo Takeuchi (1892–1968), who made great contributions to the sawfly systematics in eastern Asia.

#### Notes.

Six additional studied specimens (1 female, 5 males) from Hokkaido were not included in the type series. The female and most of the males have a longer postocellar area (more than 2 times of the lateral ocellus diameter) compared to the specimens from Honshu (mostly less than 2 times). Serrulae of the Hokkaido female are also slightly different (cf. http://www.morphbank.net/?id=693521 and Fig. 19). No clear differences were found in the structure of penis valves between the specimens from Hokkaido and Honshu.

### Empria tridens

(Konow, 1896)

http://species-id.net/wiki/Empria_tridens

Poecilosoma (Poecilosoma) tridens Konow, 1896: 54, 58. Type locality: Europe “Europa fere tota" [original description]. Lectotype female (designated in Prous et al. 2011b), DEI [examined].
Empria (Empria) caucasica Dovnar-Zapolskij, 1929: 38–39. Synonymy according to Conde (1940), see Prous et al. (2011b) for details.
Empria (Triempria) konowi Dovnar-Zapolskij, 1929: 39–40. Type locality: Russia, Sarepta. Lectotype female (designated in Prous et al. 2011b), ZISP [examined].
Empria (Triempria) gussakovskii Dovnar-Zapolskij, 1929: 40–41. Type locality: Russia, Kostroma District. Lectotype female (designated in Prous et al. 2011b), ZISP [examined].

#### Taxonomic affinities.

Belongs to *Empria longicornis* group. Morphologically the closest species is *Empria longicornis*, from which it can be distinguished in most cases by shorter antennae and more pairs of pale patches on abdominal terga (4 large and 1 small in *Empria tridens*, on terga 2–6; 3 large and 1 small in *Empria longicornis*, on terga 2–5), and by its less prominent serrulae (Fig. 22; http://www.morphbank.net/?id=578850).

#### Host plants.

*Rubus idaeus* and possibly *Rubus fruticosus* complex (Prous et al. 2011b).

#### Distribution.

Palaearctic. Specimens studied are from Belgium, Croatia, Denmark, Estonia, Finland, France, Germany, Hungary, Japan (Hokkaido), Mongolia, Russia (Amur Oblast, Kamchatka Krai, Kostroma Oblast, Leningrad Oblast, Primorsky Krai, Sakhalin Oblast, Stavropol Krai, Volgograd Oblast), Sweden, Switzerland, Turkey, Ukraine, and United Kingdom.

### Empria tridentis

Lee & Ryu, 1996

http://species-id.net/wiki/Empria_tridentis

Empria tridentis Lee & Ryu, 1996: 23. Type locality: South-Korea, Goseong-gun Hyangnobong, 38.3167N 128.3E. Holotype female, YUIC [examined].

#### Taxonomic affinities.

Morphologically, no close relatives can be identified, but in the phylogenetic analysis of the ITS and mtDNA sequences combined, the species appears as a sister of the *longicornis*-group (Fig. 40). Superficially may resemble *Empria longicornis*, from which *Empria tridentis* can easily be distinguished by tegulae, base of metatibia, trochanters, and trochantelli pale (all black in *Empria longicornis*), and by very different structure of lancets and penis valves.

#### Host plants.

Unknown.

#### Distribution.

East Palaearctic. Specimens studied are from Japan (Hokkaido, Honshu), Russia (Khabarovsk Krai, Primorsky Krai), and South-Korea.

#### Notes.

The original description of this species states that there are “a pair of large flecks on lateral portion of lst–4th tergite" (Lee and Ryu 1996), while actually no specimen studied (including the holotype) has pale patches (“large flecks") on first tergite. There is one male (NSMT018) from Honshu (Nagano) with penis valve slightly different (see http://www.morphbank.net/?id=592669) from all the other studied males, but the material is currently insufficient to decide if the specimen is aberrant or represents a separate (sibling) species.

### Empria sp. 1

#### Taxonomic affinities.

Belongs to *Empria longicornis* group. Externally it is most similar to *Empria japonica*, but penis valve is clearly distinct from all other known species of the* longicornis*-group (Fig. 36), being most similar to *Empria alpina* Benson, 1938 (e.g. http://www.morphbank.net/?id=577439). Can be distinguished from *Empria alpina* by its colouration: in *Empria *sp1 tegulae, posterior margin of pronotum, and basal 1/3 of metatibia are pale, while in *Empria alpina* these are mostly black. Distinctness of this taxon is also supported by nuclear ITS sequence data (Fig. 38).

#### Host plants.

Unknown.

#### Distribution.

Japan (Hokkaido).

#### Notes.

Because taxonomy of the *longicornis*-group is quite difficult (Prous et al. 2011b) and the corresponding female remains to be found yet, additional material is needed to describe and name this presumably new species.

## Molecular phylogenetic analyses

Bayesian analyses of the mitochondrial and nuclear sequences separately and in combination all resulted in somewhat different topologies (Fig. 38–40), with well supported differences in some cases (especially in the *longicornis* and the *immersa*-groups). However, several clades were reconstructed in all analyses with significant statistical support (posterior probability 0.95 or more). Based on these analyses, the basal split within the genus *Empria* is between *Empria candidata* and all other species (Fig. 38–40), which is consistent with the division of the genus into two subgenera, *Parataxonus* MacGillivray, 1908 (*Empria candidata*) and *Empria* s. str. (Zhelochovtsev and Zinovjev 1988; 1996; Yan et al. 2009). Monophyly of the *immersa*-group, the* longicornis*-group, and the *quadrimaculata*-group is well supported in all our analyses (Fig. 38–40). *Empria quadrimaculata* species group is proposed here for the first time for the species sharing the same type of lancets (Fig. 15–16; http://www.morphbank.net/?id=693500) and penis valves (Fig. 26–27; http://www.morphbank.net/?id=693502). A clade comprising the *longicornis*-group and the* immersa*-group, *Empria tridentis*, and *Empria takeuchii* is well supported in the analysis of nuclear ITS and in the combined analysis of ITS and the mitochondrial sequences (Fig. 38, 40). In the analysis of the mitochondrial DNA sequences, however, *Empria takeuchii* is excluded from this clade, but without significant support for any other sister-group relationships within *Empria* s. str. (Fig. 39). The sister group of *Empria honshuana*, revealed in the analyses of ITS and the combined sequences, is *Empria pallimacula* (Fig. 38, 40), but according to the mitochondrial sequences, it is *Empria excisa* (Fig. 39).

Each of *Empria japonica*, *Empria loktini*, *Empria longicornis*, *Empria immersa*, and *Empria plana* is monophyletic (as would be expected from morphology) according to the ITS sequences (Fig. 38), but not according to the mitochondrial DNA (Fig. 39). The monophyly of *Empria tridens* is supported neither by ITS nor the mitochondrial sequences (Fig. 38–39; see discussion in Prous et al. 2011b). Remarkably, *Empria* sp. 1 (USNM2051678_040) has an identical mitochondrial haplotype with one specimen of *Empria loktini* (TUZ615180), while morphology (cf. Fig. 33 and 36, see also the key) and the nuclear ITS sequences (Fig. 38) clearly differentiate these species.

**Figure 37.**
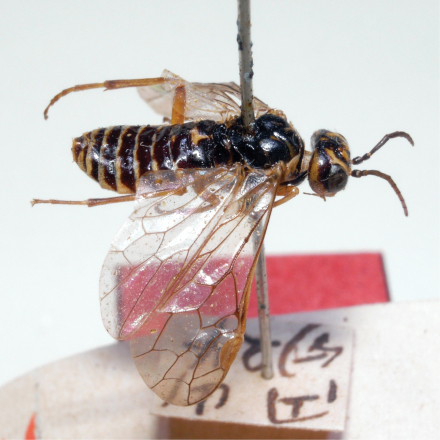
*Monsoma pallipes,* lectotype of *Poecilosoma pallipes* Matsumura, 1912, habitus in dorsolateral view, female.

**Figures 38–40.**
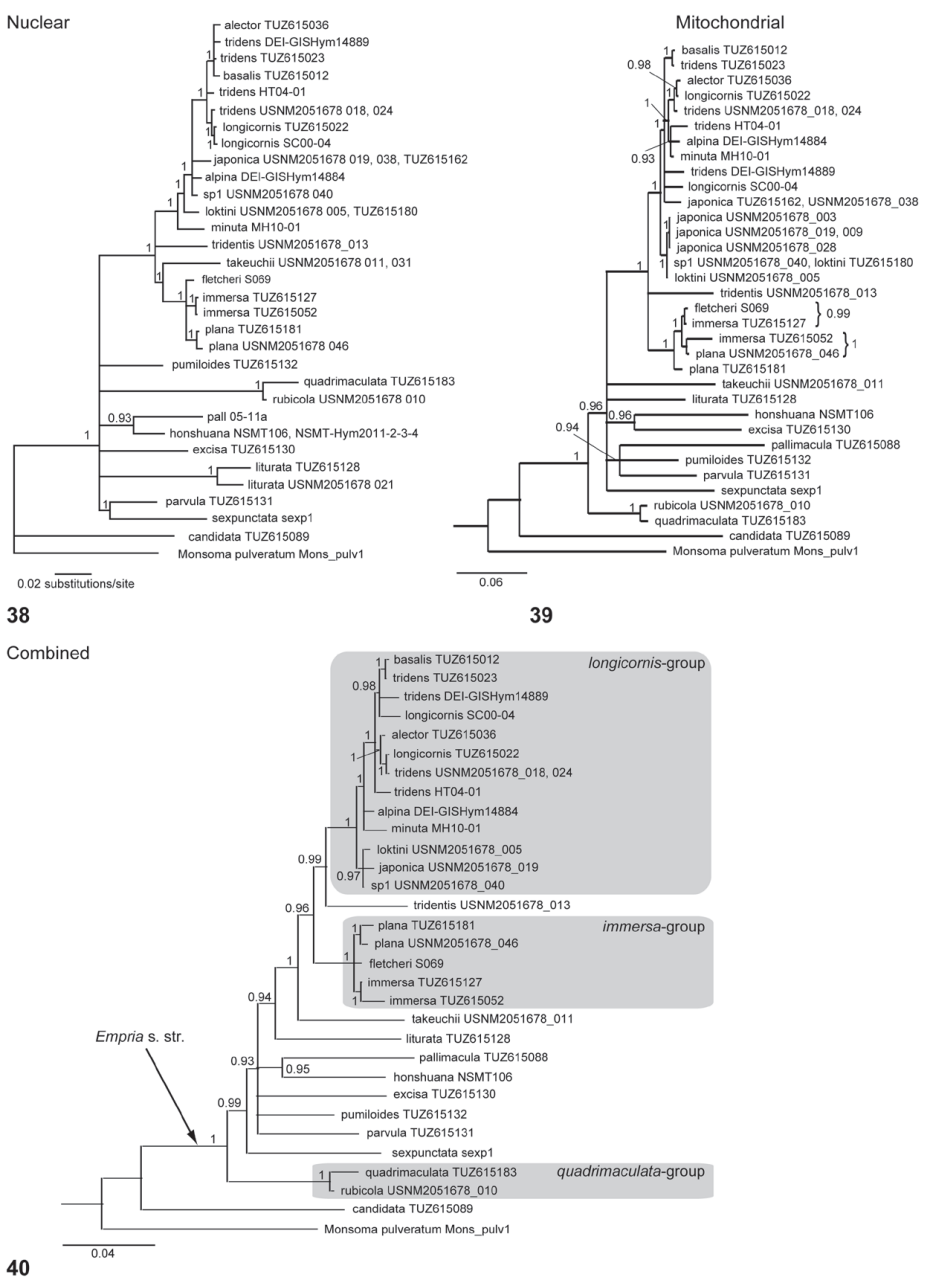
Phylogenetic analyses of the genus *Empria*. **38** Phylogeny of ITS sequences (1298–1517 bp) reconstructed using BAli-Phy (GTR + I + G[4] substitution model). Because the four independent runs of BAli-Phy produced different topologies, only clades which were found in all trees and were supported with posterior probabilities (PP) 0.9 or more are shown. Duplicate (shown behind the sequence used in the analysis) and very similar sequences (three *Empria japonica*, two *Empria tridentis*, and one *Empria rubicola*) were removed prior to analyses to reduce computation time. **39** Phylogeny of mitochondrial sequences using MrBayes (GTR + I + G[4] model; alignment length 1642 bp). Duplicate sequences (shown behind the sequence used in the analysis) were removed prior to analyses. *Empria liturata* from Japan (USNM2051678_021) was also excluded due to incomplete sequence. **40** Combined analysis of ITS (MAP alignment from BAli-Phy analysis) and mitochondrial sequences using MrBayes (GTR + I + G[4] model). *Monsoma pulveratum* was used as an outgroup. Clades with posterior probabilities (PP) less than 0.9 were collapsed in all the trees.

## Discussion

Although identification of *Empria* species using only external morphology can often be difficult, we found that females of the species reviewed here can mostly be identified without dissecting their ovipositors. Identification of the males is much less reliable without studying their genitalia because of more extensive intraspecific variation and less pronounced differences among species. The most difficult species to separate from each other on the basis of female characters are *Empria quadrimaculata* and *Empria rubicola*, the ovipositors of which appear nearly indistinguishable (Fig. 15–16). Also the external characters applied in the present key overlap considerably between them. However, because there are consistent differences in the penis valves between the two (see Fig. 26–27), they most likely represent different species.

Due to the general difficulty in identifying the *Empria* species using only external morphology, it is advisable in our opinion to leave the specimens unidentified (to avoid possible confusions in the future), especially those from the poorly studied regions (e.g. Eastern and Central Asia), as long as their identity remains problematic from external morphology and the genitalia cannot be dissected.

In addition to the 11 named *Empria* species and one presumably new but undescribed species (currently only one male is known) reported here, some additional species of the genus are likely to be found in Japan. Alpine habitats above the tree line might be inhabited by additional *Empria* species, but from there we have no samples yet.

The results of our molecular phylogenetic analyses (Fig. 38–40) significantly supported the groupings within *Empria* that could be expected from morphology (*Empria* s. str., *immersa*-group, *longicornis*-group, and *quadrimaculata*-group). Although *Empria pumiloides* was the only species from the *hungarica*-group in the current dataset, monophyly of this group is also supported by DNA data (unpublished results). The consistent affinity found between the *longicornis*-group, the *immersa*-group, and *Empria tridentis* in all our analyses (Fig. 38–40) was the only phylogenetic result not expected from morphology (though phylogenetic analyses using morphological data are still lacking). Based on the phylogenetic results presented here, we cannot draw any more definite conclusions regarding the phylogeny of *Empria*, which require, in addition to improving taxon and gene sampling, possibly also methodological advancements (e.g. using methods which take into account incomplete lineage sorting; Heled and Drummond 2010). The conflict between ITS and mitochondrial phylogenies within the *Empria longicornis* and the *Empria immersa* species groups (Fig. 38–39; see also Prous et al. 2011b) needs further study as well (e.g. sequencing 1–3 additional nuclear markers). However, we note that incongruence between mitochondrial phylogeny with morphology and nuclear phylogeny is not uncommon among closely related species, possibly because of mitochondrial introgression (e.g. Linnen and Farrell 2007; Wahlberg et al. 2009; Near et al. 2011). Another explanation, which we cannot exclude based on current data, might be incomplete lineage sorting (for a review, see Degnan and Rosenberg 2009).

## Supplementary Material

XML Treatment for Monsoma pallipes

XML Treatment for Empria candidata

XML Treatment for Empria japonica

XML Treatment for Empria honshuana

XML Treatment for Empria liturata

XML Treatment for Empria loktini

XML Treatment for Empria plana

XML Treatment for Empria quadrimaculata

XML Treatment for Empria rubicola

XML Treatment for Empria takeuchii

XML Treatment for Empria tridens

XML Treatment for Empria tridentis

XML Treatment for Empria
